# Non-surgical Retrieval of Separated Endodontic Files Using Ultrasonics and Braiding Technique: A Case Series

**DOI:** 10.7759/cureus.92415

**Published:** 2025-09-15

**Authors:** Shikha Jaiswal, Sachin Gupta, Anshika Saxena, Shivali Tyagi, Garima Verma

**Affiliations:** 1 Department of Conservative Dentistry and Endodontics, Subharti Dental College and Hospital, Meerut, IND; 2 Department of Conservative Dentistry and Endodontics, Subharti Dental College, Swami Vivekanand Subharti University, Meerut, IND

**Keywords:** braiding technique, fractured instrument, retreatment, separated file retrieval, ultrasonics

## Abstract

Managing endodontically treated teeth with canal obstructions, such as separated instruments, posts, metallic points, etc., presents a significant clinical challenge. Instrument separation during root canal therapy can compromise effective cleaning and shaping, potentially affecting the overall treatment outcome. The presence of a fractured instrument often limits access to the apical root canal region, impeding its adequate cleaning and thereby compromising the prognosis of the case. Therefore, retrieval of the separated fragment is essential to optimize clinical success. Among the various techniques available, ultrasonics has proven to be highly effective for removing fractured instruments by enabling precise navigation within complex canal anatomies. This case series details an efficient approach for the successful retrieval of separated instruments from the root canals using a combination of ultrasonic instruments, such as forceps and H-file.

## Introduction

Separation of endodontic instruments within the root canal is a common and often challenging complication encountered during endodontic treatment. This mishap can obstruct access to the apical third of the canal, impede proper cleaning and shaping, and potentially compromise the long-term prognosis of the tooth [1]. Despite the advances in endodontic instruments, particularly with the introduction of nickel-titanium (NiTi) rotary files, which offer improved flexibility and resistance to fracture, instrument separation continues to be reported, with prevalence ranging from 0.25% to 10%. Instrument fracture can result from torsional failure, cyclic fatigue, or manufacturing defects and may occur even in the hands of experienced clinicians [2].

To retrieve or bypass the instrument is a clinical decision often dictated by the amount of remaining tooth structure and sound dentin, the availability of techniques like ultrasonics and guided endodontics, and largely the clinician's ability. Various techniques and instruments have been described in the literature for instrument retrieval, including forceps, broaches, H-files, chemical solvents, hypodermic surgical needles, and specialized retrieval kits such as the Masserann kit (Micro-Mega, Besançon, France), Cancellier Extractor (SybronEndo, Orange, CA, USA), and Meitrac Endo Safety System (Meisinger, Denver, CO, USA) [3], with guided endodontics being the most recent approach [4,5].

Among all, the most popularly used technique is with ultrasonic devices, which, when used in conjunction with magnification through a dental operating microscope, has emerged as a highly effective method for retrieving separated instruments. Ultrasonic energy can be precisely directed to loosen the fragment from the canal walls through vibration, facilitating its dislodgement [6]. Reported success rates of ultrasonic techniques range from 67% to as high as 95%, depending on factors such as canal anatomy, fragment location, and operator skill [7,8].

This case report presents three cases in which separated instruments were successfully retrieved from root canals using a combination of ultrasonics, magnification, and braiding technique, thereby emphasizing the use of an appropriate combination of conventional and contemporary strategies for managing this challenging endodontic complication. The novelty or significance of this case series is the combination of ultrasonic retrieval with the braiding technique. Conventional instrument retrieval kits would result in excessive dentin loss. Minimally invasive techniques like ultrasonics and braiding technique conserve dentin and are much more predictable clinically.

## Case presentation

Case 1

**Figure 1:**
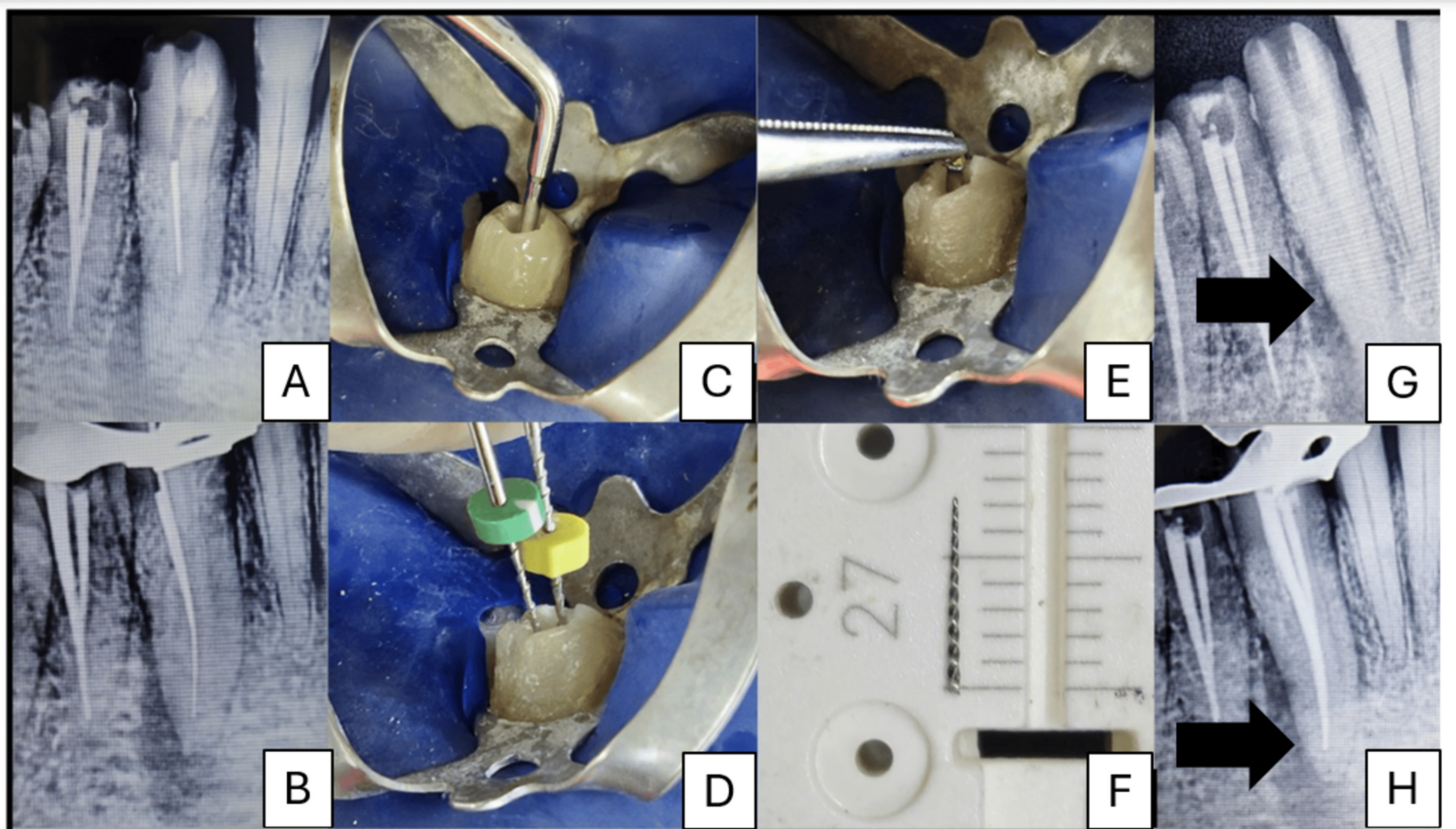
File retrieval from tooth 43 using ultrasonics and braiding technique. (A) Pre-op IOPA of 43 with a broken instrument. (B) Missed lingual canal negotiated. (C) Ultrasonic tip T3 is used to loosen the separated file. (D) Braided technique with H-files (#20, #35). (E) Instrument retrieved from the canal. (F) 7-mm-long retrieved file fragment. (G) Radiograph confirming complete file retrieval. (H) Post-obturation radiograph. IOPA: intraoral periapical

A 50-year-old male patient presented to the outpatient Department of Conservative Dentistry and Endodontics with a complaint of persistent discomfort in the lower right canine region and a history of incomplete root canal treatment. Medical history was non-contributory. On clinical examination, 43 was tender on percussion. Radiographic examination revealed an approximately 7-mm-long separated endodontic instrument lodged in the middle third of the canal (Figure 1A). The canal presented with mild curvature apical to the tip of the separated instrument with a coexistent periapical pathology.

Patient's consent was taken for the treatment plan, which included retreatment with file retrieval using ultrasonics by creating a staging platform using specialized troughing tips (modified Gates-Glidden (GG) drills) under magnification (10×), followed by the revision of shaping and obturation. Temporary restoration was removed under rubber dam isolation, and straight-line access was established to the fractured instrument while simultaneously locating a missed canal (Figure 1B). GG drills were used to create a staging platform. Ultrasonic cutting tip T1 (DTE series, Woodpecker Dr. Talal's Endodontic kit, Guilin, China) was utilized at a frequency of 24-33 kHz and a power setting of 7, in wet mode and "E" function, to trephine the dentin around the coronal end of the instrument (Figure 1C). Non-cutting ultrasonic tip T3 was used around the coronal aspect of the separated instrument to cut through the dentin, creating space which led to the loosening of the separated file. Since the file could not be retrieved with ultrasonics alone, the braided technique was used, which involves the simultaneous insertion of three small Hedström files (ISO #10, #10, and #15) alongside the separated fragment, gently maneuvered to surround the fragment circumferentially within the canal (Figure 1D). Once in place, the three files were twisted/braided together, effectively entangling the broken segment within the entwined instruments. With gentle counterclockwise traction, the separated instrument was successfully retrieved in one piece along with the braided files (Figure 1E, 1F). After radiographically verifying the complete removal of the separated instrument (Figure 1G), both canals were shaped using the crown-down technique till F2 ProTaper (Dentsply Sirona, Charlotte, NC, USA), and intracanal medicament was placed. At one-week recall, the patient was asymptomatic, and obturation was done using F2 ProTaper gutta-percha cones, followed by composite resin restoration (Figure 1H).

Case 2

**Figure 2:**
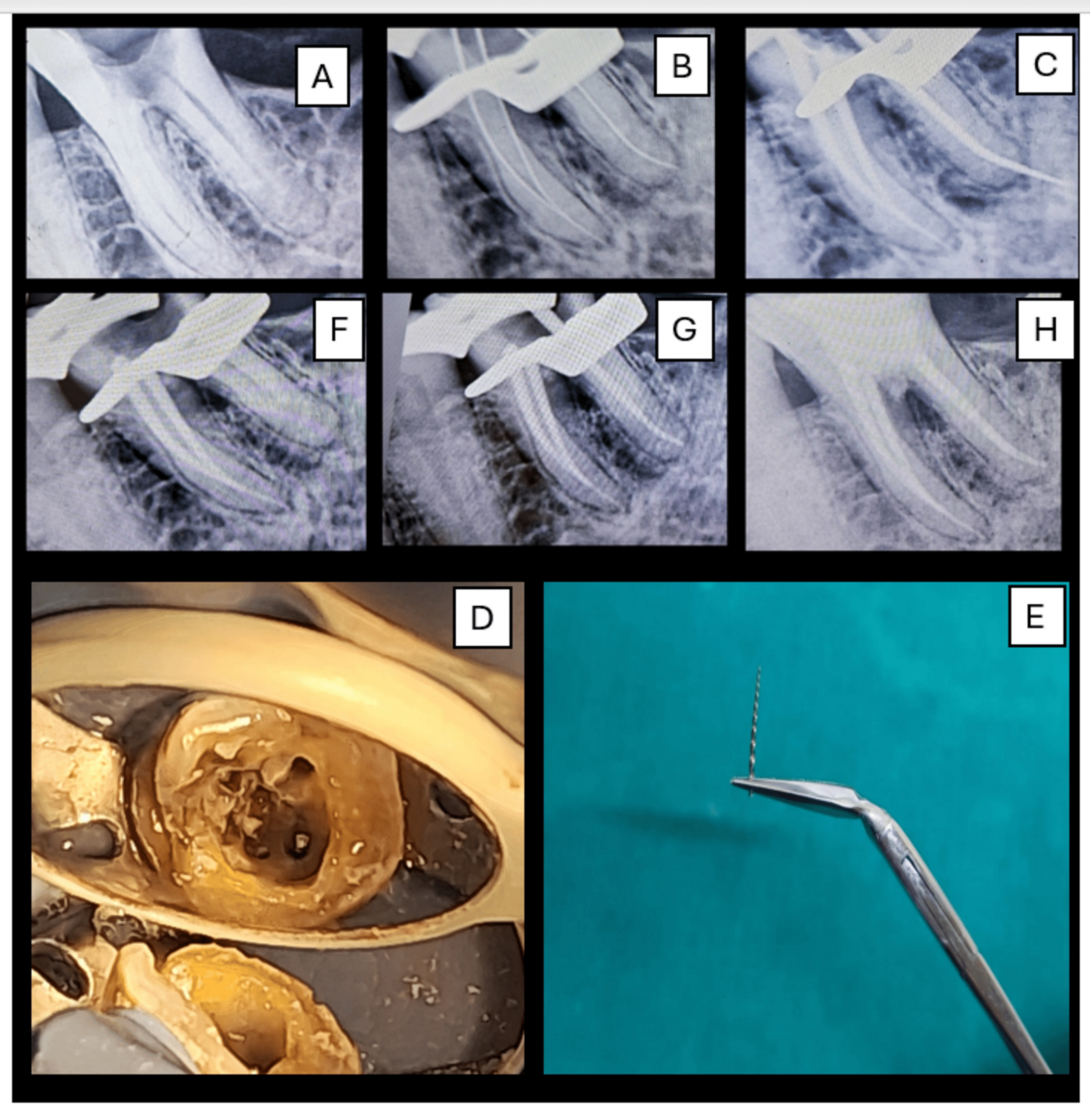
File retrieval from tooth 46 using ultrasonics. (A) Pre-op radiograph showing deep caries in #46 with periapical radiolucency. (B) Working length determination. (C) Separated ProTaper Gold file due to misjudged working length in the distal canal. (D) Ultrasonic agitation with the E15 tip to loosen the fragment. (E) Retrieved file fragment using Steiglitz forceps. (F) Radiograph confirming complete file retrieval. (G) Post-obturation radiograph. (H) Final radiograph showing furcal perforation repair with MTA and Abgel. MTA: mineral trioxide aggregate

A 39-year-old male patient presented with the chief complaint of continuous pain in the lower right back tooth region for one week. Clinical and radiographic examination revealed deep caries in #46, which was tender to percussion (Figure 2A). Pulp vitality testing indicated a prolonged response suggestive of a diagnosis of symptomatic irreversible pulpitis with symptomatic apical periodontitis. Endodontic treatment was planned and initiated under rubber dam isolation. After access cavity preparation, mesiobuccal and mesiolingual canals were prepared to a 20/.07 taper using ProTaper Gold rotary files and obturated with F1 gutta-percha cones and a bioceramic sealer using the single-cone technique.

During the preparation of the distal canal, the working length was misjudged, leading to the separation of the ProTaper file. Radiographic examination showed a fractured file extending beyond the apex and the coronal end being visible at the canal orifice (Figure 2B, 2C). After obtaining informed consent from the patient, file retrieval was attempted. Under magnification and illumination, Woodpecker E15 ultrasonic tips were carefully used in a brushing motion around the fractured file to loosen it from the surrounding dentin (Figure 2D). Once the fragment was adequately loosened and visible at the orifice, Steiglitz forceps (Medesy, Maniago, Italy) were gently introduced to grasp and retrieve the exposed fragment successfully (Figure 2E). After confirming complete removal radiographically and clinically (Figure 2F), cleaning and shaping of the distal canal was completed, followed by obturation with an F2 ProTaper cone (Figure 2G). During retrieval and subsequent instrumentation, a furcal perforation was noticed, which was managed promptly. Hemostasis was achieved using saline irrigation, and the perforation site was managed by placing an internal matrix of Abgel, followed by sealing the defect with mineral trioxide aggregate (MTA) (Figure 2H).

Case 3

**Figure 3:**
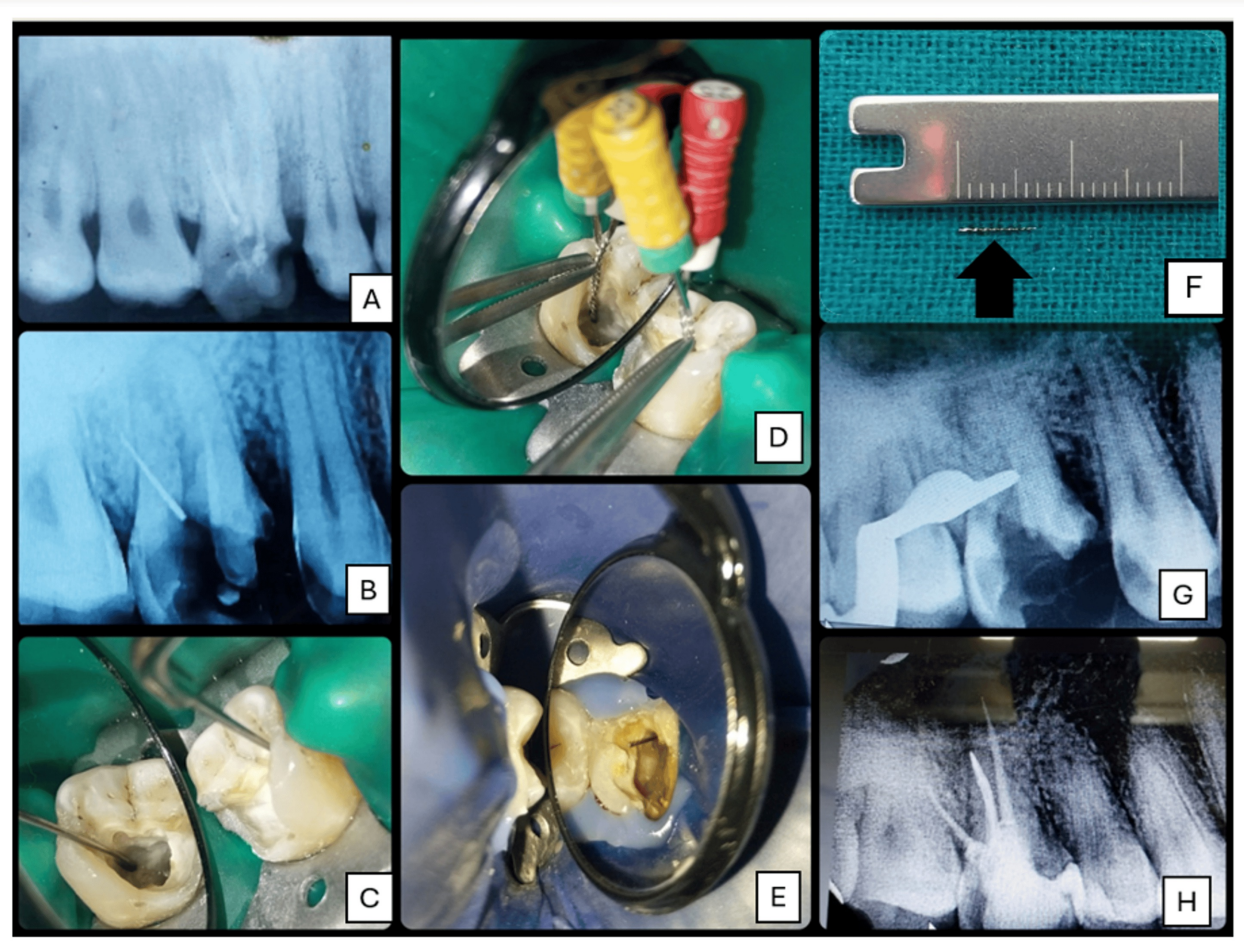
File retrieval from tooth 16 using ultrasonics and braiding technique. (A) Pre-op IOPA. (B) IOPA post-gutta-percha removal and confirmation of the separated instrument in the distobuccal canal. (C) Use of ultrasonic cutting tip T1 and non-cutting tip T3 for access refinement. (D) Braiding technique with H-files #20 and #25. (E) Retrieved instrument visible in chamber. (F) Retrieved instrument for scale (approx. 7 mm). (G) IOPA confirming complete file retrieval and patency. (H) Post-obturation IOPA IOPA: intraoral periapical

A 30-year-old female patient was referred to the Department of Conservative Dentistry and Endodontics for retreatment of the maxillary right first molar (#16). Clinical examination revealed that the tooth was tender to percussion and buccal palpation. The preoperative intraoral periapical (IOPA) (Figure 3A) showed an incompletely obturated and restored 16, with a separated instrument in the distobuccal canal extending from the coronal to the middle third of the canal and slight widening of the lamina dura around the palatal root. A final diagnosis of symptomatic apical periodontitis was made, and instrument retrieval was planned after obtaining the patient's consent.

After rubber dam isolation, gutta-percha was removed from all canals using the sequential use of H-files #10, #15, and #20 and chemical solvent (xylene). An IOPA post-gutta-percha removal confirmed the presence of a separated instrument in the distobuccal canal (Figure 3B).

The access was refined using a Piezo ultrasonic scaling system (Coltene Biosonic S1L Portable Scaler, Altstätten, Switzerland) with ultrasonic cutting tip T1 (Figure 3C) (DTE series, Woodpecker Dr. Talal's Endodontic kit) under 10× magnification. Subsequently, a non-cutting ultrasonic tip T3 was used peripherally to the instrument. Although the instrument was loosened, it could not be fully retrieved, so the braiding technique was used by entwining #20 and #25 H-files around the separated instrument (Figure 3D), and after repeated attempts, eventually the separated instrument was retrieved from the canal (Figure 3E, 3F). An IOPA confirmed canal patency post-retrieval (Figure 3G). The canals were biomechanically shaped with F1 ProTaper (mesiobuccal and distobuccal) and F2 ProTaper (palatal). Intracanal medicament was packed, and the patient was recalled after one week. In the following visit, the tooth was asymptomatic and obturated with corresponding ProTaper gutta-percha cones using bioceramic sealer, followed by post-endodontic restoration (Figure 3H).

## Discussion

The retrieval of a separated endodontic instrument remains one of the most technically demanding procedures in endodontics. The difficulty in removal depends on numerous factors, the most critical being the location of the separated instrument in the canal. Retrieval is easier and less time-consuming when the instrument is in the coronal third than in the middle or apical third due to accessibility. A separated instrument can compromise canal debridement and disinfection, potentially affecting treatment prognosis.

Success rate for the retrieval of separated instruments ranges from 55% to 79% [9]. While advancements in technology, such as ultrasonic devices, dental operating microscopes, retrieval kits, or guided endodontics [10], have significantly improved success rates, it is the clinician's patience and experience that often determine the final outcome.

Lack of knowledge and skills and overzealous attempts to retrieve the instrument may lead to excessive dentin loss, creation of ledges, etc., that affect the long-term prognosis of the tooth. Hence, the fundamental principle in such situations should be to approach the case methodically, taking time to assess all variables, including the canal anatomy, location of the fragment, visibility, and accessibility, before embarking on the treatment protocol [2].

Clinicians should formulate a step-by-step protocol, starting with careful coronal pre-flaring and ultrasonic troughing under high magnification, and while multiple attempts are required during file retrieval, one should be mindful of not making any additional iatrogenic errors during the process. Since the process may require multiple visits, clear communication, reassurance, and realistic timelines help in maintaining trust and compliance with the patient. Each case is unique, and progress may be slow. However, resisting the urge to rush enhances tactile sensitivity and decision-making, reducing the risk of further complications [11].

The staging platform is a critical preparatory step in the retrieval of a separated endodontic instrument. It involves the careful modification of the canal coronal to the separated instrument to create a flat and centered platform around the fragment. The purpose of the staging platform is multi-fold. It improves visualization, provides unobstructed access, limits unnecessary dentin removal, preserves root structure, and reduces the risk of perforation, allowing for better control during ultrasonic activation, reducing the chance of pushing the fragment apically [12].

After achieving accessibility to the coronal end of the separated instrument, retrieval can be attempted by ultrasonics, which has become one of the most effective and widely accepted strategies [13]. The technique involves the use of ultrasonic tips activated at high frequency under magnification to dislodge the fragment through vibration and controlled dentin removal. Intracanal corrosion and debris may bind the file fragment mechanically to the surrounding dentin. Ultrasonic energy, when applied to the exposed portion of a separated instrument, creates microvibrations, thus disrupting the debris and loosening the fragment. These vibrations break up debris and disrupt any mechanical interlocking or corrosion, bonding the fragment to the canal walls. This can allow the fragment to be either withdrawn passively or flushed coronally with irrigation [14].

Another conventional and minimally invasive technique that can be utilized for instrument retrieval is the braided technique. In this technique, clinicians rely on tactile feedback to engage and remove a separated instrument fragment lodged deep within the canal. In this procedure, two or three Hedström files are placed alongside the fragment within the canal. The first file engages the fragment by "screwing" it into its flutes, while the remaining files intertwine to form a braided structure. This braided construct enhances gripping force as all files are simultaneously withdrawn, aiming to extract the captured fragment. Hedström files with their fluted design are preferred for optimal engagement. This technique proves particularly valuable when fragments lie deep within the canal and are not readily visible, but success hinges on operator skill and the careful assessment of canal anatomy to avoid complications [15]. This technique becomes all the more relevant after the use of ultrasonics and slight loosening of the file, as has been discussed in the above cases. Long-term follow-up of six months to one year is recommended to evaluate the success rates.

## Conclusions

The successful retrieval of separated instruments in endodontics hinges on a number of critical factors: the clinician's unwavering patience and experience, a well-curated armamentarium, and the judicious use of techniques like braiding and the latest technology like ultrasonics. It should be noted that these are merely illustrative case reports using a combination of conventional braiding technique and contemporary ultrasonics. The outcome of such cases largely depends on the skill and experience of the operator and should not be generalized as comprehensive evidence of efficacy. Hence, further studies, preferably larger case series or controlled studies, are necessary to confirm the outcomes. In essence, instrument retrieval is not just a technical procedure but a testament to the endodontist's skill, preparation, and perseverance. When these elements align, even the most daunting retrieval cases can be transformed into predictable and successful outcomes.
